# Central retinal artery occlusion secondary to presumed traumatic carotid artery dissection in a healthy child

**DOI:** 10.1186/s40942-022-00411-2

**Published:** 2022-08-19

**Authors:** Thiago José Muniz Machado Mazzeo, Raimunda Cristina Mendonça Freire, Luciano Fuzzato Filho, Cleide Guimarães Machado, André Marcelo Vieira Gomes

**Affiliations:** 1 Retina and Vitreous Department, Suel Abujamra Institute, São Paulo, Brazil; 2 Retina and Vitreous Department, University of São Paulo (USP), São Paulo, Brazil

**Keywords:** Antithrombotic, Carotid artery injury, Cetinal artery occlusion

## Abstract

**Purpose:**

To describe a rare case of a 13 years-old healthy child that presented CRAO secondary to carotid artery dissection, which occurred after a neck rotation movement.

**Methods:**

Case report with prospective literature review.

**Patients:**

One patient described in the case report.

**Results:**

Not applicable.

**Discussion/conclusion:**

Internal carotid artery dissection is a rare condition, specially in children, that can lead to serious cerebral-ocular ischemic events. It may occur due to direct vessel trauma or spontaneously. Prompt imaging screening is of paramount importance because early antithrombotic treatment or surgical intervention may significantly reduce the incidence of devastating ischemic events, such as stroke or central retinal artery occlusion.

## Introduction

Vertebral and Internal carotid arteries are critical cervical arteries. Any injury that may eventually occur to these vascular structures can lead to vessel thrombosis and dissection, leading to complications such as cerebral ischemia, stroke, blindness, or death [1].

Internal carotid artery dissection (ICAD) results from disruption of the intima of the arterial wall, leading to blood intrusion into the arterial wall, which forms an intramural hematoma. The hematoma can compress the true lumen of the vessel, causing functional stenosis, occlusion, and predisposing to thrombus formation. The classic triad signs of ICAD include pain in the ipsilateral neck, head, and orbital regions; a (partial) Horner syndrome; and cerebral or retinal ischemia. However, not all ICAD patients present with these classic signs, and ocular manifestations may sometimes be the only initial findings [1, 2].

Ocular ischemia secondary to ICAD, may range from ischemic optic neuropathy and ocular ischemic syndrome to branch/central retinal artery occlusions [3]. Central retinal artery occlusion (CRAO) is a rare ophthalmic emergency that presents with sudden, severe, painless monocular visual loss. In children, this condition is even rarer, and in most cases, a systemic cause can usually be identified when carefully investigated [4].

Internal carotid artery dissection may be caused by blunt trauma, but it can also occur spontaneously, especially in patients with predisponent systemic conditions such as fibromuscular dysplasia and Marfan syndrome [5]. Unfortunately, given its rarity and nonspecific symptoms, ICAD is difficult to make a prompt diagnosis.

In addition, extreme neck movements (hyperextension, rotation, or flexion) may eventually lead to carotid arterial injury in various ways, as will forwardly be described in this article.

This report aims to describe a rare case of a 13 years-old healthy child that presented CRAO secondary to carotid artery dissection, which occurred after a neck rotation movement. To the best of our knowledge, there are very few reports in the literature describing this condition.

## Case report

A 13 years old, healthy male patient presented with sudden unilateral visual loss in the right eye 12 h after cervical lateral flexion. The patient related persistent ipsilateral cervical pain right after neck moviment and his mother stated that the child had the constant habit of doing such neck movements (popularly called “neck cracking”). There is no history of previous ocular-systemic disease or trauma.

**Fig. 1:**
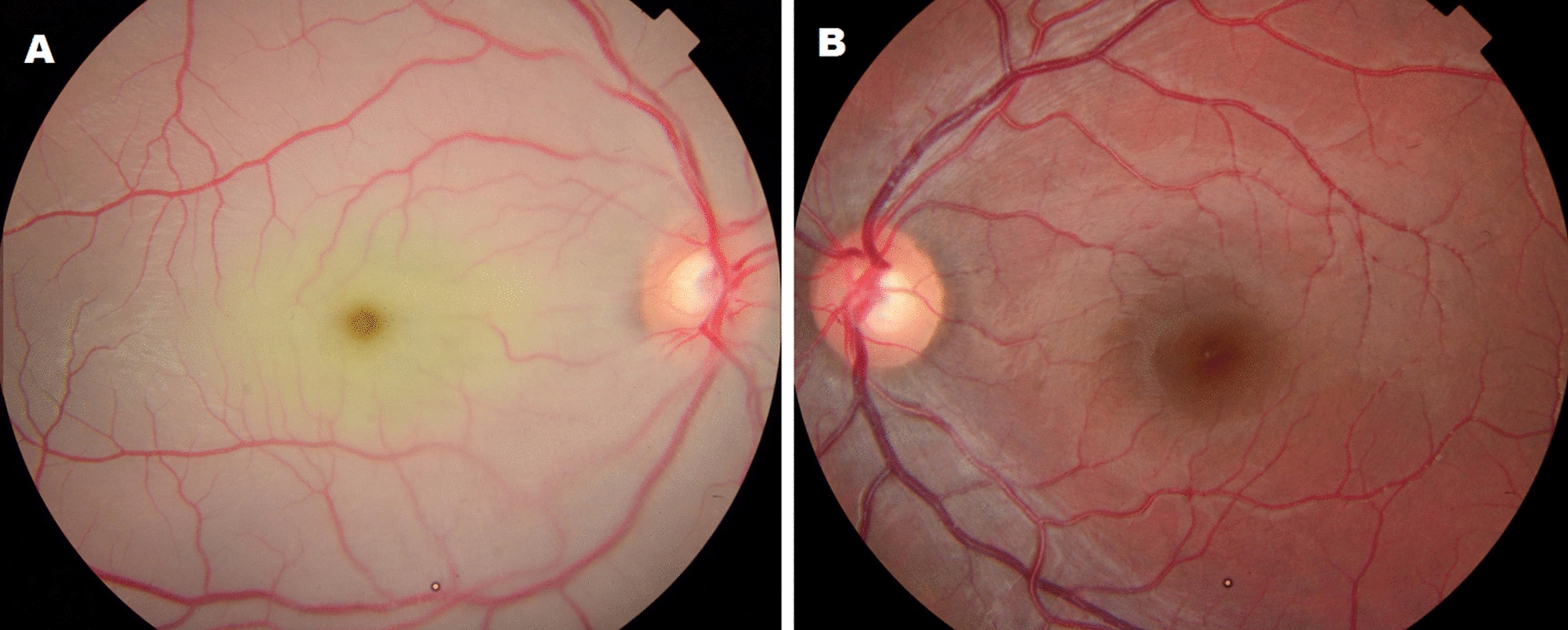
Colored Retinopraphy. **A** OD fundus demonstrating diffuse retinal pallor with a cherry-red spot in the macula. **B** OS presenting an unremarkable fundus examination

The patient’s best-corrected visual acuity (BCVA) was light perception in the right eye (OD) and 20/20 in the left eye. He had a normal anterior segment examination on slit-lamp biomicroscopy on both eyes. Fundus examination in OS was unremarkable, while OD revealed diffuse retinal pallor and a cherry-red spot in macular region (Fig. 1).

**Fig. 2:**
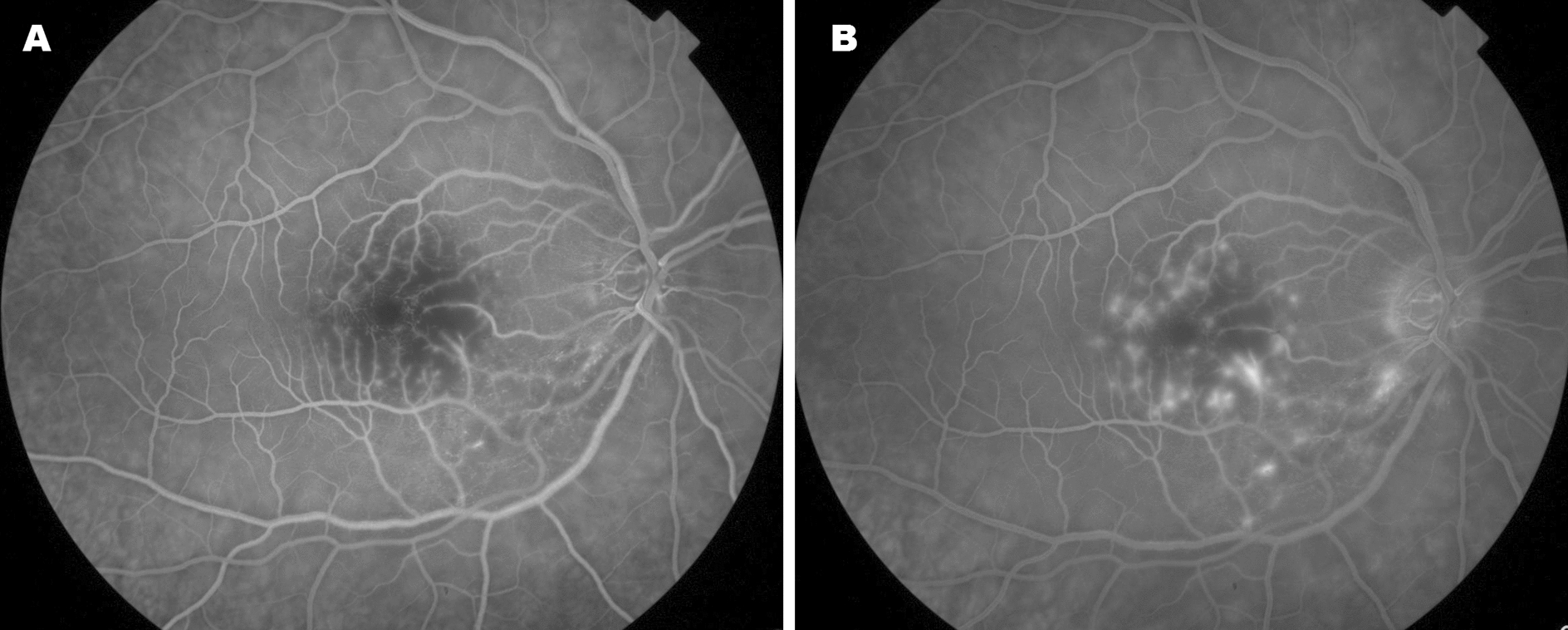
Fluorescein Angiography (FA) **A** Venous phase of FA (34 s) demonstrating hypofluorescence in the perimacular region due to capillary non-perfusion **B** Late venous phase (4 min) showing posterior hyperfluorescent (leakage) spots due to capillary damage

The fluorescein angiography exam presented a mild delay in the arm-to-retina time, and hyperfluorescence (leakage) was present diffusely among perimacular capillaries (Fig. 2).

**Fig. 3:**
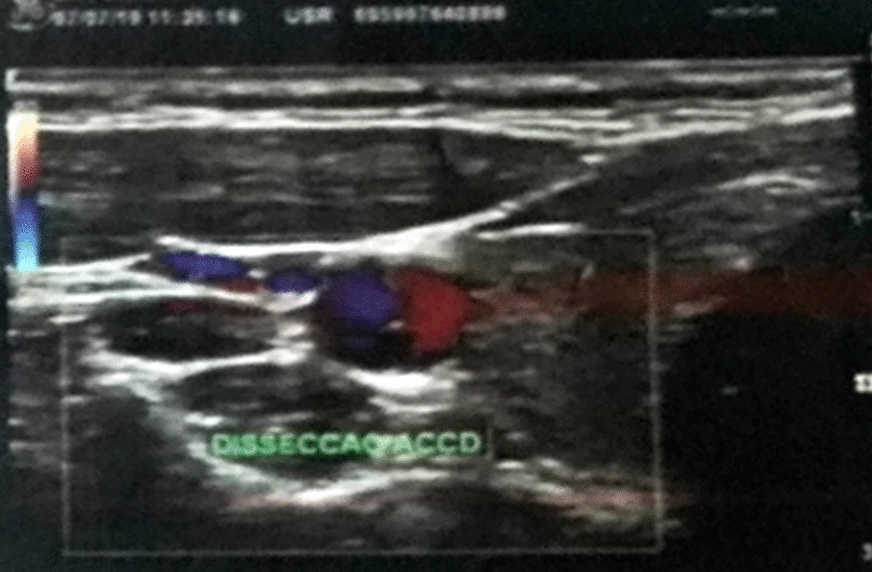
Ecocollordoppler image of the right common carotid artery. The exam demonstrated dissection of the vessel’s intimal layer, creating a false lumen

Ecocollordoppler examination (Fig. 3) demonstrated an intraluminal flap in the right common carotid artery, suggestive of arterial dissection. A comprehensive systemic evaluation was carried out, and no abnormal condition was identified.

The patient was evaluated by the vascular surgery department, and oral antithrombotic (acetylsalicylic acid) treatment was initiated. After 30 days, BCVA in visual OD was fingers count 30 at cm (OS maintained 20/20). Carotid arterial injury responded successfully to clinical treatment, even though severe visual impairment in OD was maintained.

## Discussion

Carotid arterial dissection is described as being an uncommon cause of retinal artery occlusion, and its incidence in adults after blunt head and neck injury is estimated at 0.3–0.67%. In children, this injury seems to be significantly less common, estimated at 0.03%. The pathophysiology of ICAD is not completely understood, but patients with connective tissue diseases and concomitant arterial anomalies are at higher risk [5, 6].

Carotid artery dissection may also develop without previous blunt trauma or predisponent condition. In fact, previous studies proposed mechanisms for carotid arterial injury related to neck movement, such as described by the patient in this report. Vigorous neck movement may lead to carotid arterial injury in various ways, such as neck hyperextension, rotation, or flexion, causing stretching damage to the vessels. These movements may put the contralateral carotid at risk, as it can be stretched against the second and third cervical vertebral bodies. The carotid artery may also be injured by the styloid process during sudden rotation or compressed by the mandible’s angle during hyperflexion [6].

Vessel stretch may result in intimal injury, creating the potential for vessel dissection or intramural thrombus formation. This way, severe thromboembolic events may proceed carotid lesions, which may range from a devastating ischemic stroke to arterial retinal occlusions [2]. We believe our patient had a significant (but not complete) ICA dissection that lead to a turbulent bloodflow, predisposing thrombus formation. Probably due to its size not being so large to cause an ophthalmic artery occlusion, nor too small to cause a BRAO, it traveled through bloodstream, thromboembolizing central retinal artery. Patients with carotid injury may be asymptomatic upon initial presentation, with the majority of ischemic events or neurologic symptoms occurring within the first seven days [7].

In the literature, there are studies proposing other vessel injury mechanisms, as Raser JM et al. demonstrated that a longer styloid process could predispose carotid injury compared with case-matched controls [8]. Stroke and mortality rates increase according to the vessel injury’s progression and worsening. This way, In the absence of contraindications, It is widely accepted that early antithrombotic therapy may reduce the incidence of ischemic events. Nonetheless, it has been observed that despite medical intervention, some cases of CAI can progress in severity requiring surgical intervention [9].

The relative rarity of carotid arterial injury, paired with the need for prompt diagnosis, poses a clinical challenge when attempting to identify these patients rapidly. To date, there is no guideline as to which imaging modality is best suited for routine screening of CAI, but most authors advocate the use of computerized tomography angiography (CTA) as an initial screening method, despite four-vessel Digital subtraction angiography (DSA) being the gold standard modality. However, DSA carries more risk and is less available in daily clinical practice [10].

On the other hand, Doppler ultrasonography may not be adequate as an initial screening tool, as it has poor sensitivity, but it may be useful as a follow-up imaging modality [11].

## Conclusion

Retinal artery occlusion secondary to carotid artery dissection in healthy children is a very rare condition that needs prompt evaluation and treatment because CAD may lead to devastating cerebral-ocular ischemic events. Despite there is little consensus, most authors suggest CTA as the first screening modality, even though it has variable sensibility and specificity, compared to DSA, which is the gold-standard screening exam. Doppler ultrasonography is also described to have questionable sensitivity, but it may be useful as a follow-up imaging modality.

Antithrombotic agents are indicated in mild-moderate carotid injuries; however, close follow-up is needed since injury progression may occur, requiring surgical intervention.

## Data Availability

Not applicable.

## References

[1] Song JX, Lin XM, Hao ZQ, Wu SD, Xing YX (2019). Ocular manifestations of internal carotid artery dissection. Int J Ophthalmol.

[2] Biffl WL, Moore EE, Offner PJ, Burch JM (2001). Blunt carotid and vertebral arterial injuries. World J Surg.

[3] Mokhtari F, Massin P, Paques M, Biousse V, Houdart E, Blain P (2000). Central retinal artery occlusion associated with head or neck pain revealing spontaneous internal carotid artery dissection. Am J Ophthalmol.

[4] Ratra D, Dhupper M (2012). Retinal arterial occlusions in the young: systemic associations in Indian population. Indian J Ophthalmol.

[5] Chamoun RB, Mawad ME, Whitehead WE, Luerssen TG, Jea A (2008). Extracranial traumatic carotid artery dissections in children: a review of current diagnosis and treatment options. J Neurosurg Pediatr.

[6] Lee TS, Ducic Y, Gordin E, Stroman D (2014). Management of carotid artery trauma. Craniomaxillofac Trauma Reconstr.

[7] Burlew CC, Biffl WL, Moore EE, Barnett CC, Johnson JL, Bensard DD (2012). Blunt cerebrovascular injuries: redefining screening criteria in the era of noninvasive diagnosis. J Trauma Acute Care Surg.

[8] Raser JM, Mullen MT, Kasner SE, Cucchiara BL, Messe SR (2011). Cervical carotid artery dissection is associated with styloid process length. Neurology.

[9] Bromberg WJ, Collier BC, Diebel LN, Dwyer KM, Holevar MR, Jacobs DG (2010). Blunt cerebrovascular injury practice management guidelines: the Eastern Association for the Surgery of Trauma. J Trauma.

[10] van Asch CJ, Velthuis BK, Rinkel GJ, Algra A, de Kort GA, Witkamp TD (2015). Diagnostic yield and accuracy of CT angiography, MR angiography, and digital subtraction angiography for detection of macrovascular causes of intracerebral haemorrhage: prospective, multicentre cohort study. BMJ.

[11] Mutze S, Rademacher G, Matthes G, Hosten N, Stengel D (2005). Blunt cerebrovascular injury in patients with blunt multiple trauma: diagnostic accuracy of duplex Doppler US and early CT angiography. Radiology.

